# The Memory T Cell “Communication Web” in Context with Gastrointestinal Disorders—How Memory T Cells Affect Their Surroundings and How They Are Influenced by It

**DOI:** 10.3390/cells11182780

**Published:** 2022-09-06

**Authors:** Annkathrin Knauss, Michael Gabel, Markus F. Neurath, Benno Weigmann

**Affiliations:** 1 Medical Clinic 1, Research Campus, University Hospital Erlangen, Hartmannstr. 14, 91052 Erlangen, Germany; 2 Medical Immunology Campus Erlangen, Friedrich-Alexander Universität Erlangen-Nürnberg, 91054 Erlangen, Germany; 3 Medical Clinic 1, University Hospital Erlangen, Ulmenweg 18, 91054 Erlangen, Germany; 4 Deutsches Zentrum Immuntherapie (DZI), 91054 Erlangen, Germany

**Keywords:** memory T cells, IBD, Crohn’s disease, ulcerative colitis, colorectal cancer, interactions, immune cells, metabolites, microbiota, etrolizumab, vedolizumab, ozanimod

## Abstract

Gut-related diseases like ulcerative colitis, Crohn’s disease, or colorectal cancer affect millions of people worldwide. It is an ongoing process finding causes leading to the development and manifestation of those disorders. This is highly relevant since understanding molecular processes and signalling pathways offers new opportunities in finding novel ways to interfere with and apply new pharmaceuticals. Memory T cells (mT cells) and their pro-inflammatory properties have been proven to play an important role in gastrointestinal diseases and are therefore increasingly spotlighted. This review focuses on mT cells and their subsets in the context of disease pathogenesis and maintenance. It illustrates the network of regulatory proteins and metabolites connecting mT cells with other cell types and tissue compartments. Furthermore, the crosstalk with various microbes will be a subject of discussion. Characterizing mT cell interactions will help to further elucidate the sophisticated molecular and cellular networking system in the intestine and may present new ideas for future research approaches to control gut-related diseases.

## 1. Introduction

Gut-related disorders represent a pervasive issue all over the world. A highly prominent example is the presence of inflammatory bowel diseases (IBD) which can be further defined as Crohn’s disease (CD), ulcerative colitis (UC), or, if no clear differentiation is possible, indeterminate colitis [1]. Patients affected by those disorders suffer symptoms like abdominal pain, diarrhoea, and psychological distress [1,2,3]. Furthermore, colorectal cancer (CRC) can occur as a subsequent event of IBD and is determined as one of the most prevalent cancer forms globally [4,5,6]. Decades of research discovered multiple aspects associated with the pathogenesis of IBD and CRC. External factors such as specific dietary habits like a high-fat, high-sugar diet, better known as a “Western-style” diet, contribute to IBD and CRC, in addition to internal factors like genetics or the dysbiosis of the intestinal microbiota [6,7,8]. Overall, disturbances in the immune system homeostasis and inappropriate immune responses appear to be major factors of the IBD causality [8]. Gastrointestinal disorders are multifactorial diseases and various body components must be considered. Yet, explicit mechanisms promoting disease development are still unknown. Considering the ~7 million IBD patients worldwide and the increasing incidence and prevalence rate, root cause analysis in the context of IBD and CRC pathogenesis is crucial in order to both prevent IBD and CRC development and treat affected patients [1]. In this context, T cells and their subsets are of special interest. Their role in the development and maintenance of gut-related diseases has been studied intensively over the past years. This review will focus on a special type of T cell characterized by their immunological memory properties, and therefore named memory T cells (mT cells). Depending on their expression of surface markers, gene transcription, location, and role in immunity, they can further be classified mainly into central memory cells (TCM), effector memory cells (TEM), and tissue resident memory cells (TRM) [9]. Other cell types exhibiting a memory phenotype like memory regulatory cells or stem cell memory T cells will not be discussed in the present review [10,11,12]. Generally, memory T cells provide long-term immunosurveillance and protection against reoccurring pathogens. After detecting familiar harmful microorganisms, mT cells can act rapidly and more effectively, as after the first infection inducing counteractive measures in order to eliminate pathogens within hours after exposure [13,14]. However, aside from their function as potent and protective sentinels preventing re-infections, adverse events related to mT cells have been reported as well. Recently, enhanced migration of TCM cells to the lungs of mice demonstrating an allergic airway inflammation was observed using an asthma mouse model. This finding resulted in the hypothesis that inhibiting TCM infiltration might come with benefits in the treatment of lung inflammation [15]. In context with allergic asthma, it was also reported that long-lived pathogenic Th2 TRMs can produce Th2 cytokines and drive allergen-induced disease relapses [16]. Moreover, mT cells, especially TRMs, have been negatively associated with IBD and cancer. Zundler et al. described a pathogenic role of TRMs in patients with CD and UC and evaluated it in experimental colitis models [17]. Furthermore, TRMs were identified as a major source of pro-inflammatory cytokines in patients with active CD [18].

## 2. Characterisation of Memory T Cells

Upon antigen presentation by macrophages or dendritic cells, naïve T cells become activated, proliferate, and differentiate. As a next step, they either migrate to inflamed tissue to eliminate pathogens themselves or further stimulate B cells to differentiate and produce antigen-specific antibodies. Additionally, another fraction (up to 10%) of the primed T cells can develop, providing long-term memory and immunosurveillance. Based on their phenotypic and functional properties, they can further be defined as TCMs, TEMs, or TRMs [19,20,21].

## 3. Central and Effector Memory T Cells

Sallusto et al. initially defined TCMs and TEMs as memory T cell subsets in 1999 by analysing human peripheral blood. The C-C chemokine receptor type 7 (CCR7] was designated to distinguish TCMs with a high expression of CCR7 (CCR7^high^) from TEMs demonstrating low levels of CCR7 (CCR7^low^) [22]. Together with CD62L, also known as L-selectin, which is strongly expressed on TCMs and appears less on TEMs, CCR7 is essential for homing of secondary lymphoid organs (SLOs) and therefore contributes to the recirculation of TCMs through SLOs [20,21]. Furthermore, TCMs and TEMs can either be CD4^+^ or CD8^+^, with the latter demonstrating a CD45 RA^−^ CCR7^+^ phenotype for TCMs and a CD45 RA^−^ CCR7^−^ phenotype for TEMs. Naïve T cells express both CD45 RA and CCR7 and effector T cells express only CD45 RA without the CCR7 receptors [23]. However, there are also TEMs re-expressing CD45 RA (CD45^+^ CCR7^−^) indicating a cytotoxic potential and relevance in antiviral immunity [24]. Both CD4^+^ TEMs and TCMs are positive for the CD45 isotype CD45R0, whereas naïve CD4 cells express CD45 RA [23]. As prevailing for T cells, the expression of receptors and adhesion molecules is exquisitely complex and with respect to cellular interaction in constant transition, which was already demonstrated in the 1990s [22,25]. Therefore, characteristic phenotypical descriptions mostly apply to resting cells [26].

Characterising TEMs and TCMs is an ongoing process since these cell populations are defined by their heterogeneity. Multiple more markers such as CXCR3 or CX3CR1 can be used to unravel their subsets, including so-called peripheral memory cells [27,28].

Regarding the formation of TCMs and TEMs, different ideas and models have been expressed over the years. Starting with the differentiation of naïve T cells to stem cell memory T cells, TCMs might be generated that can further develop into TEM cells. Other models describe effector T cells or specific memory precursor cells as upstream cell types, from which memory T cells can evolve. Here, effector T cells can differentiate into either short-lived effector cells expressing low levels of the interleukin 7 receptor, or into memory precursor effector cells with high levels of IL7-R and the potential to form long-lived memory T cells [29,30]. The various models and theories are well reviewed and illustrated by Liu et al., 2020 [31]. Based on studies from Akondy and Youngblood, the effector T cell to memory T cell transition model is most likely due to a preserving effector-cell-like epigenetic signature demonstrated by the authors [32,33]. Amongst other stimulatory interactions leading to the differentiation and maintenance of TCMs and TEMs, cytokines play a major role. Especially IFNγ, IL2, IL7, IL15, IL15Rα, and IL21 are repeatedly reported in the context of mT homeostasis and regulation [12]. IL2 can act through binding to either the dimeric IL2R consisting of the γ chain (γc) and IL2Rβ, which is also termed as CD122, or the trimeric receptor form, having CD25 additionally to γc and CD122 [34,35,36]. CD127 linked to γc serves as a receptor for IL7 whereas IL15 induces its signalling by either binding to the dimeric IL2 receptor or to a trimeric complex formed by γc, CD122, and IL15 Rα [12,37].

In general, both TCMs and TEMs have cytotoxic properties upon antigen stimulation. However, TEMs are far more efficient when it comes to eliminating their targets. It was demonstrated that TEMs express markedly higher levels of the death mediators perforin, granzyme B, and death receptor FasL compared to TCMs [22,38]. Even though TCMs have a weaker capacity to directly act against targets, they can generate increased levels of IL2. Subsequently, IL2 can trigger T cell proliferation and can activate and stimulate dendritic cells to produce IL12. TEMs have proven properties in synthesizing high levels of IFN-γ, IL4, and IL5 [22,39].

## 4. Tissue Resident Memory T Cells

In the early 21st century, researchers began to assume that besides recirculating TCMs and TEMs, another cell type with memory properties exists remaining in the tissue over a long time [40,41,42]. Recently, this memory T cell fraction has been well established and termed as tissue resident memory cells. Comparably with TEM cells, TRM cells express CD62L and CCR7 only marginally [43,44]. TRMs are mainly defined by their phenotypical features enabling their tissue retention. The surface markers CD69 and CD103 are predominantly used to differentiate TRMs from their recirculating mT cell counterparts. CD69 expression on T cells marks their antigen-experienced status since the lectin’s expression is upregulated after the T cell activation. However, constitutive CD69 expression is restricted to resident cells [45,46]. Nevertheless, TRM subsets without CD69 exist, making this surface molecule an indefinite TRM marker [47]. Still, CD69 can interfere with the sphingosine-1 phosphate receptor (S1PR1] as it is an antagonist of S1P—the ligand of S1PR. S1PR is secreted by endothelial cells and T cells as well. It is responsible for luring T cells out of the tissue back into the circulation as the cells follow the S1P gradient with high S1P levels in the blood and low levels in the tissue. Consequently, CD69 complexes with S1P1 initiating the internalization and degradation of S1PR1 from the cell surface and subsequently diminished tissue egress [48,49,50,51,52]. CD103, also named αE integrin can pair with the integrin β7 and serves as a receptor for E-cadherin expressed on epithelial cells [49]. This adhesive interaction contributes to TRMs tissue retention and further explains the positioning of a fraction of those cells within the epithelium [49,53]. Comparably to CD69, CD103 expression is not an exclusive TRM surface marker considering that there are also TRMs lacking this integrin on their surfaces [54]. Overall, many additional cell surface markers have been linked to TRMs such as CD44, CD49a, and the lymphocyte function-associated antigen-1 (LFA-1], however, their expression can vary depending on the tissue and organ they are located in. For instance, CD49a together with β1 integrins also known as CD29 pairs with collagen of the extracellular matrix mediating tissue retention. It belongs to the major memory population in the lung and is further prevalent in the skin [55,56,57]. LFA-1 is associated with liver-located TRMs [58].

In general, TRMs can develop and differentiate from antigen-triggered memory precursor effector cells under the influence of multiple factors. In contrast to terminal effector cells that arise from activated naïve T cells and express the killer cell lectin-like receptor G1 (KLGR1], precursor cells are characterized by low levels of KLRG1 and enhanced levels of IL-7Rα, also named CD127 [59,60]. Two cytokines are reported to have major impacts on the further effector-to-memory T cell transition and TRM cell maintenance in the tissue: TGF-β as it can both diminish the presence of the transcription factors Eomes and T-bet and can promote the expression of CD103, and IL15 mediating TRMs’ long time survival [61,62]. Moreover, TNFα, IL33, and interferons type 1 might negatively affect the expression of the Krüppel-like factor 2 (KLF2] which is a transcription factor leading to further downstream events, such as the missing transcription of S1PR1 (a target gene for KLF2] [63,64]. Additionally, an upregulation of the CD69 surface markers is associated with those cytokines [48]. Contrary to the transcription factors KLF2, T-bet, and Eomes, which are similarly downregulated upon TRM cell development, Hobit (a homolog of Blimp-1], Runx3, and Notch are upregulated [48,65].

TRM cells are reported to be present in various body sites and tissue compartments like the brain, lung, liver, and the gastrointestinal tract [62]. As for the latter, TRM cells are mainly located in the lamina propria or the epithelium, exhibiting either a CD4^+^ or a CD8^+^ phenotype with or without the expression of CD103, and can persist in the intestine for years [66,67]. TGF-β is an important player driving the differentiation of either the CD103^+^ or the CD103^−^ occurrence of intestinal TRMs. CD103^+^ cells require TGF-β signalling and are widely scattered within the tissue, whereas CD103^−^ cells develop independently of TGF-β and are predominantly found in the lamina propria-associated inflammatory microenvironment [68]. In addition to TGF-β, IFN-β and IL12 have been identified as key regulators of the differentiation of the CD103^−^CD69^+^ intestinal TRM population [69]. TGF-β signalling controls the TRM formation and maintenance in the gut by both anticipating the migration of effector T cells from SLOs to the intestine via downregulating α4β7, and by inducing αEβ7 and CD69 [70].

As reported by Senda et al., TRM cells can build early in life and their prevalence and distribution remain steady over the years [71]. Upon activation by re-exposure to a familiar antigen, TRM cells can react rapidly. Their effectiveness is characterized by the secretion of cytokines and chemokines recruiting several immune cells such as dendritic cells (DCs), natural killer cells, and TEMs/TCMs to the site of infection and stimulating nearby lymphocytes. Thus, TRMs can act as sentinels [48]. Moreover, TRMs can directly eliminate danger-associated patterns due to their cytotoxic properties, including the generation of granzyme B and perforin [72].

TRMs also can rejoin the circulating pool upon reactivation. As Fonseca et al. showed, intestinal TRMs can undergo retrograde migration back to circulation and have the potential to differentiate into TCM cells. These ex-TRMs remain epigenetically embossed for homing their original tissue and reacquiring their TRM phenotype and characteristics [73,74].

## 5. Memory T Cells and Their Role in Gut-Related Diseases

The role and effects of mT cells in context with gut-related disorders are ambivalent, expressing their light and dark side. From the mT cell pool, TRM cells and TRM-like cells predominantly have been reported to have an impact on various colitis models and IBD. In this regard, Lamb et al. observed enhanced expression levels of IFNγ and TNFα from CD4^+^αEβ7^+^ lymphocytes in UC compared with CD4^+^ cells lacking the αEβ7 cell marker. Evaluating cohorts of healthy control subjects and active UC patients, they further noted that the expression of αEβ7 came along with an upregulated Th1 and Th17 cytokine production and a diminished expression of regulatory T cell-related markers. These findings postulate that the presence of CD4^+^αEβ7^+^ in the colon has pro-inflammatory properties and might contribute to UC pathogenesis [75]. Comparable results were published 2 years later by Bishu et al. when they analysed human colon samples from CD patients as well as from healthy controls [18]. Their results deciphered CD4^+^ TRMs as the major T cell source of mucosal TNFα in patients with CD. Furthermore, the production of IL17A was elevated compared to control samples. When suppressing the gene *PRDM1* (also known as Blimp-1], which is a highly expressed transcription factor in CD4^+^ TRM cells, the induction of IL17A, TNFα, and additional inflammatory cytokines was alleviated. Taken together, Bishu et al. attributed an important role to TRMs in CD and hypothesized therapeutic benefits in manipulating and targeting TRMs in IBD [18]. Two more colitis studies from Sasson et al. and Zundler et al. negatively associated TRM cells with colitis. The group of Zundler et al. reported an accumulation of pro-inflammatory CD4^+^ TRMs in IBD patients and correlated the presence of TRMs with the development of occurring flares in the patients. In an additional mouse study, a double knockout of the genes Hobit and Blimp-1 resulted in an attenuated disease outcome in various colitis models [17]. Whereas Zundler et al. focused on CD4^+^ TRM cells, Sasson et al. investigated the role of CD8^+^ TRMs in immune checkpoint inhibitor-colitis and demonstrated that this mT cell subsets activation correlates with the severity of colitis. Moreover, aligned with previously mentioned studies, they confirm that TRMs express high levels of IFNγ [76]. However, aside from the repeatedly reported pro-inflammatory role of TRMs in gut-related disease, there are also published data contradicting their negative role. Roosenboom et al. detected lower ratios of both CD4^+^ and CD8^+^CD103^+^ cells in patients at IBD diagnosis and active inflammation remained at low levels during follow-up analysis [77]. Reduced numbers of CD8^+^ TRMs in CD and UC patients were also reported by Noble et al., suggesting that promoting rather than suppressing TRM functions might improve the intestinal barrier, function leading to an overall favourable outcome [78].

TRM cells are further associated with colorectal cancer. Noble et al. recently tested the hypothesis that TRMs are involved in the development of parainflammation and tumorigenesis. Analysing biopsies from CRC patients and healthy controls indicated a clear reduction of CD8^+^ and CD4^+^ TRM cells in the CRC patient samples. Further data linked disturbances in microbiota homeostasis to the depletion of TRM cells and therefore impaired tumour immune surveillance [79]. Considering previous data, chemoradiotherapy can stimulate the TRM cell activation and expand the tumour-reactive TRMs, expressing CD103 resulting in the best histological responses after therapy [80]. Moreover, successful immunotherapy of CRC is associated with an increased number of anti-tumour CD8^+^ TRMs [81].

Even though identifying and measuring mT cells is possible, one must keep in mind that a genuine quantification of those cells is still challenging and might not represent actual cell numbers present in vivo. It is suggested that mT densities are several times bigger than detected [47].

## 6. The Memory T Cell Networking System

### 6.1. Interaction with Microbiota—Regulatory Proteins and Metabolites

The large intestine harbours multiple variants of commensal bacteria, viruses, fungi, and other types of microorganisms that are summarized as the intestinal microbiota. Their presence is crucial for the host by not only providing a physical barrier against harmful trespassers but also by producing metabolites that are useful to both the intestinal epithelium and the immune system [82]. Disturbances in the microbiota composition and homeostasis are widely reported to contribute to gut-related disorders.

As mentioned previously, Bishu et al. provided evidence that CD4^+^ TRMs are a primary TNFα resource in CD patients [18]. Subsequently, they linked those findings to other studies outlining that mucosal TNFα-producing T cells can interact with the enteric flora. It was previously demonstrated by Hegazy et al. that a large fraction of CD4^+^ circulating mT cells and gut-resident T cells are reactive to the microbiota and secrete the proinflammatory cytokines TNFα, IL2, and IL17A [83]. However, it must be considered that IL2 and IL17A are indefinite proinflammatory markers. The inhibition of IL17A by antibodies or an IL17A deficiency can also induce and impair colitis as reported in both mouse and human studies [84]. IL2 is further required for not only the development but also the long-term survival of regulatory T cells [85]. Additionally, it can impede TH17 polarization and can limit the inflammation during immune responses [86]. Hegazy et al. chose bacterial species predominant in the gut and/or associated specifically with IBD and analysed T cell responses toward those bacteria. They reported 2- to 4-fold increased levels of CD4^+^ memory T cells in inflamed tissue sites from IBD patients compared to non-affected tissue sites and healthy controls. Overall, it was suggested that under steady-state conditions dendritic cells stimulate gut-resident CD4^+^ T cells at low levels by recognizing luminal antigens triggering the secretion of cytokines beneficial for intestinal homeostasis. Contrary, in IBD existing with dysbiotic changes and more frequent contact with micro-organisms, overreacting T cells negatively influence proinflammatory cytokine production [83]. Furthermore, Sasson et al. theorized that commensals or pathogenic microorganisms directly evoke a CD8^+^ TRM response, resulting in enhanced IFNγ signalling and tissue activation [76].

**Figure 1:**
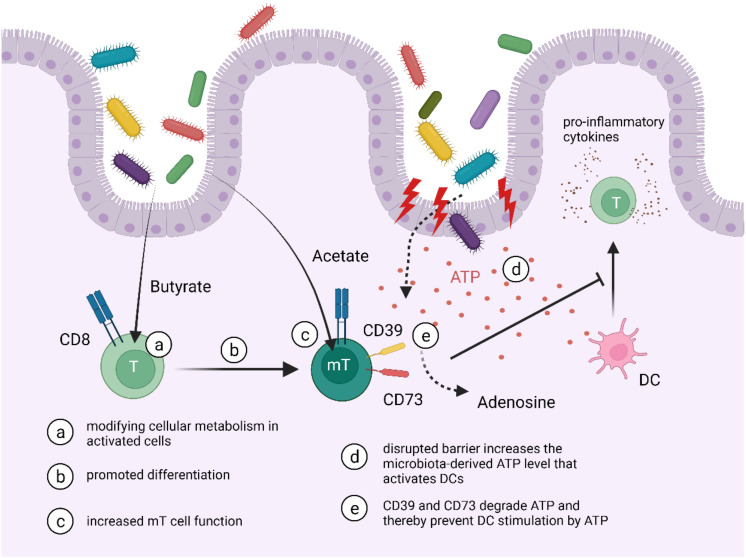
*Impact of microbiota on mT cells.* The presence of microbiota is essential for the CD8^+^ T cell to mT cell transition. SCFAs like acetate and butyrate contribute to mT cell differentiation and can enhance their memory property. The co-expression of CD39 and CD73 on gut resident mT cells can induce the degradation of ATP released by mucosa infiltrating bacteria. It might therefore attenuate the activation of DCs and possibly reduce pro-inflammatory responses.

Bachem et al. further observed interactions between microbiota and mT cells. In their published data they exhibit evidence that the presence of microbiota is pivotal for the activated CD8^+^ T cells to memory CD8^+^ T cell transition. Also, microbiota-provided metabolites such as short-chain fatty acids (SCFAs) can increase CD8^+^ T cell memory properties. Butyrate specifically as a SCFA was contributing to the memory T cell differentiation by interfering, modifying, and newly interconnecting the cellular metabolism in activated CD8^+^ T cells, including uncoupling the Krebs cycle from glycolytic input [87] (see Figure 1). Not only butyrate but also acetate has been set in context with mT cells as they can take up acetate resulting in enhanced memory T cell function [88] (see Figure 1). However, it has to be mentioned that those data were not related to the intestinal compartment explicitly.

Noble et al. analysed antigen-specific responses toward commensal bacteria in IBD by challenging PBMCs with selected commensal strains for 7 days [78]. Even though the results were highly variable, they discovered the CD4 T cell response as a predominant memory reaction and further that numbers of CD8 T cell responses were reduced in IBD patients compared to healthy controls. When correlating numbers of CD8 TCM responses from blood analysis towards the commensal strains with CD8 TRM responses obtained from biopsies, they discovered a significant positive correlation. Overall, they hypothesised that IBD is associated with diminished systemic CD8 T cell responses towards selected bacterial strains, which leads to a TRM deficiency in the mucosa of the large intestine. Furthermore, Noble et al. observed that TRMs in the gut co-express key functional markers of T_reg,_ namely CD39 and CD73. As they discuss in their publication, CD39 and CD73 can degrade extracellular ATP released by bacteria and can thereby attenuate the activation of dendritic cells induced by the presence of ATP [78,89,90]. Since DCs can generate and stimulate effector T cells and therefore induce pro-inflammatory responses, promoting TRMs and their cross-linkage may improve IBD-related disturbed barrier function inflammation [78,91] (see Figure 1). Nonetheless, DCs are also able to induce regulatory T cells preventing and limiting inflammatory responses toward low levels of pathogenic antigens [91]. On these grounds, investigating the interaction of TRMs with DCs in context with IBD is vital.

### 6.2. Interaction with Other Cell Types and Compartments

Research from Bottois et al. suggested the existence of functionally different CD8 T cell subsets with a memory background in the bowels according to their expression of CD103 and KLRG1 markers. Additionally, their results indicated that the proportion of those TRM subsets is associated with the peculiarity of the inflammation in the intestine. Moreover, CD103^+^CD8^+^ TRMs indicated increased responsiveness to TCR triggering in contrast to CD103^−^ cells, and those cells also actively contributed to immune responses in the intestinal mucosa. In addition, they found out that CD103 TRMs expressed enhanced levels of IL22, IL26, and CCL20 (Th17-related cytokines) when comparing cells isolated from CD patients with control patients. These results underline the relevance of TRMs in context with IBD. Regarding the modified expression levels of TH17-related cytokines, they relied on previous findings in their discussion [92]. IL22 has already been demonstrated to be upregulated in intestinal inflammation in CD patients and is an important cytokine stimulating the barrier integrity of the intestine and promoting human β-defensin expression. This indicates an indirect interaction of TRM cells with defensin (which are antimicrobial peptides) secreting Paneth cells [92,93]. In addition, CCL20 expression is altered in IBD. Kaser et al. reported a several-fold increase in both CD and UC but not in non-IBD colitis [94]. CCL20 can further attract mT cells and other cells expressing its receptor CCR6 like CD4 T cells, T_regs_, and B cells that can be both beneficial in promoting antimicrobial activity and unfavourable in respect of promoting inflammation by secreting pro-inflammatory cytokines, such as TNFα, and further attract new effector cells like KLRG1^+^CD8^+^ TRMs [92]. Single-cell studies from Corridoni et al. suggested that IL26^+^ cells could arise from TRM cells directly, that CD8^+^ cells producing IL26 are increased in UC and that the tissue expression of IL26 correlated with the inflammation. Although the induction of IL26 demonstrated positive effects in the context of epithelial damage in mice with acute DSS- induced colitis and may have protective properties in acute inflammation, Corridoni and his colleagues assumed a different role in chronic inflammation [95].

Research from Ferreira et al. addressed the interaction of regulatory T cells (T_reg_) with TRMs in various infection models [96]. As a result, they identified a direct connectivity of those cells with respect to TRM cell development. As T_regs_ are allured to sites of local inflammation by their chemokine receptor CXCR3, they can promote the generation of CD8^+^ TRMs in a T-bet dependent manner. A lack of type 1 T_regs_ not only diminishes the amount of TRMs in tissues such as the duodenum and the small intestine but also enhances the overall pathogen load upon an induced infection with *Eimeria vermiformis* which is known as a protozoan parasite infecting the epithelial cells in the murine small intestine. Overall, Ferreira et al. indicated that T-bet promotes the expression of CXCR3, leading to a recruitment of T_regs_ to the site of infection and the production of bioavailable TGF-β1 correspondingly, which leads to the TRM cell development [96]. Although this study is not directly linked to IBD or CRC, further literature proposes a relevant role of TGF-β1 in the intestinal immune homeostasis as TGF-β1 biallelic loss-of-function mutation is associated with early-onset IBD [97]. Additionally, elevated expression levels of FoxP3 and TGF-β1 were detected in CRC patients [98]. The mentioned studies do not clearly connect mT cells to IBD and CRC, however, they might offer a starting point for further research approaches.

TEM cells are also of interest regarding cell–cell interactions. Recent findings from McDaniel et al. outlined a direct interaction of TEM cells with myeloid cells such as DCs and macrophages, triggering a transcriptional program that works independently from PRR activation and leads to proinflammatory responses. By investigating the underlying pathways both in vivo and in vitro, they discovered a crucial role of CD40- together with TNF receptor signalling. They discussed the ability of CD40L- and TNF-expressing TEMs to mimic microbial ligands and activate myeloid cells initiating NFκB and mitogen-activated protein kinase pathways, resulting in a pro-inflammatory cytokine release. By blocking this pathway, McDaniel et al. were able to inhibit the TEM-induced innate inflammatory cytokine flooding in addition to the autoimmune pathology in the absence of microbes, highlighting possible targets to interfere with and counteract diseases associated with self-reactive memory T cells [99].

As mentioned above (chapter 4), CD8^+^CD103^−^ TRMs in the intestine depend on the presence of IFN-β and IL12 which can be produced and secreted by inflammatory monocyte-derived APCs [69]. Furthermore, TGF-β signalling is required for CD103^+^ TRMs in the intestine [68]. Circulating monocytes can produce and release IL-10 that can act in an autocrine signalling loop and can induce the release of TGF-β, as demonstrated in lung tissue by Thompson et al. Subsequently, TGF-β upregulates CD103 on T cells [100]. Another study identified monocytes as a required cell type for the maintenance but not generation of lung resident TRMs. However, the related underlying mechanisms are still unknown [101].

An overview of the interactions of mT cells with other cell types and the microbiota is provided in Table 1.

## 7. Therapeutical Approaches

The field of IBD therapies offers a wide range of non-biological and biological drugs targeting and interfering with various parts of the complex pathology of IBD. However, side effects, lacking long-term improvements, and failing patient responsiveness towards specific treatment underline the ongoing need for new therapeutical approaches [102].

**Figure 2:**
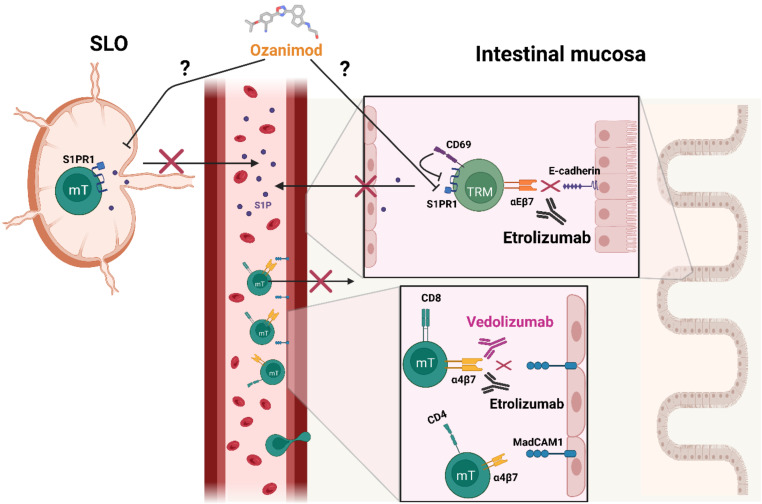
Possible effects of various drugs on mT cells. Vedolizumab can block the α4β7 integrin and can therefore inhibit the migration of T cells from the bloodstream into the intestinal mucosa, ameliorating inflammation. Etrolizumab acts not only on the α4β7 integrin but also interferes with the αEβ7:E-cadherin interaction, possibly impairing the tissue retention of TRMs. Ozanimod targets S1PR1 receptors and might therefore anticipate the egress of mT from lymph nodes into the circulation but might also contribute to and synergize the CD69-mediated tissue retention in the mucosa.

Vedolizumab is a gut-selective humanized monoclonal antibody and an integrin antagonist. It can interact with the α4β7 integrin, blocking the migration of inflammatory cells to the intestinal tissue. These properties are used for the treatment of UC and CD [102]. Analysed data from the GEMINI long-term safety study (a randomized, placebo-controlled trial investigating the long-term efficiency of Vedolizumab) demonstrated promising results regarding the effectiveness of Vedolizumab in IBD treatment [102,103,104]. Since Vedolizumab binds to peripheral memory CD4^+^ T lymphocytes with high specificity but also to memory CD8^+^ T lymphocytes, gut-selective homing of these cell types is negatively affected [105,106]. Based on these findings, one can assume a possible negative role of mT cells in IBD (Figure 2).

Another therapeutical humanized antibody-based drug is Etrolizumab—an agent proven to be operative in UC and CD patients [107]. It not only blocks the α4β7 integrin but also the αEβ7:E-cadherin interaction associated with mT cells, as Etrolizumab is an anti-β7 specific integrin antibody [108]. Since the αEβ7:E-cadherin interaction is important for TRMs in terms of tissue retention, Etrolizumab might directly interfere with TRMs. It was also indicated that a blockage induced by Etrolizumab reduced the accumulation of CD8^+^ and CD4^+^ Th9 cells in the inflamed intestine and that the majority of CD8^+^ αEβ7 T lymphocytes are TRM cells as they additionally express the TRM indicating marker CD69 [109] (see Figure 2).

Aside from the antibody-based therapeutical Vedolizumab and Etrolizumab, an oral applicable drug called Ozanimod is of interest in the treatment of not only multiple sclerosis but also IBD. It is a sphingosine 1-phosphate receptor modulator targeting the receptor subtypes 1 and 5 with a high affinity, reducing immune cell infiltration by preventing lymphocyte egress from SLOs [51,110]. Phase 2 and 3 studies provided evidence for clinical improvements upon the treatment with Ozanimod as treated UC patients demonstrated a higher rate of remission and higher clinical response compared to placebo groups [111,112]. Moreover, CD patients receiving the drug for 12 weeks demonstrated better endoscopic, histological, and clinical disease outcomes [113]. Even though those studies were not linked to mT, given their phenotypical characteristics and properties in interacting with S1PRs, it offers new possibilities for future research approaches to further elucidate the roles and impacts of mT in IBD (see Figure 2).

## 8. Concluding Remarks

The intestinal environment and organization are extremely complex. Mechanisms, interactions, and suggested signalling pathways described in this review do not represent the overall picture. Still, memory T cells are a reasonable and possibly underestimated cell population that must be considered in context with gut-related diseases. They are originally determined to rapidly protect various tissue sites from re-infections induced by familiar pathogens. However, their role in chronic diseases such as IBD remains unclear and ambivalent as research suggests both beneficial and disadvantageous effects of mT cells in gut-related disorders. Interactions with multiple cell types have been reported. Targeting mT cells and therapeutic approaches affecting these cells come with risks and pitfalls as it may also adversely influence beneficial cells of the immune system. Thus, further investigations are necessary to elucidate and identify the specific mT cell function in IBD and CRC. Deciphering the controversial properties of mT cells will further improve the administration of both already approved and prospective medications.

## Figures and Tables

**Table 1:** Overview of proposed interactions of mT cells with other cell types and the microbiota.

*mT Cell Subset*	*Interacting with*	*Cytokines/* *Mediators Involved*	*Type of Interaction*	*Impact on Inflammation*	*Reference*
***CD39^+^ CD73^+^ TRMs***	DCs	ATP	TRMs can degrade ATP released by bacteria preventing DC activation	anti-inflammatory	[78,89,90]
***CD103^+^ TRMs***	Paneth cells	IL22	TRMs release IL22 leading to Paneth cell stimulation and release of antimicrobial peptides	anti-inflammatory	[92,93]
***CD103^+^ TRMs***	CCR6^+^ cells	CCL20	CCL20 expressed by TRMs can attract CCR6^+^ cells	dichotomic	[92]
***TRMs***	T_regs_	TGF-β1	Recruited T_regs_ lead to production of bioavailable TGF-β1 production and development of TRMs	under pro-inflammatory conditions	[96,97]
***TEMs***	DCs and macrophages	CD40L, TNFα	TEMs expressing CD40L and TNFα activate myeloid cells	pro-inflammatory	[99]
***TRMs***	Monocytes	IL10, TGF-β	IL10 stimulates monocytes to secrete TGF-β leading to upregulation of CD103	—	[100]
***CD4^+^ TRMs***	Microbiota (bacterial species associated with IBD)	TNFα, IL2, IL17A	Upon microbiota sensing, TRMs secrete pro-inflammatory cytokines	pro-inflammatory	[83]
***CD8^+^ TRMs***	Commensals/pathogenic microorganisms	IFNγ	Microorganisms directly evoke TRMs leading to IFNγ signalling	pro-inflammatory	[76]
***CD8^+^ TRMs***	Microbiota	SCFAs (butyrate, acetate)	Microbiota can induce the CD8^+^ T cell to CD8^+^ TRM transition and increased memory properties	—	[87,88]

## Data Availability

Not applicable.
